# Doxycycline inhibits MMP-2 retinal activity and modulates the angiogenic process *in vitro* and *in vivo*

**DOI:** 10.3389/fcell.2025.1561250

**Published:** 2025-03-31

**Authors:** María Lina Formica, María Constanza Paz, María Victoria Vaglienti, Paula Virginia Subirada, Yamila Fernández, Mariana Belén Joray, José Domingo Luna, Pablo Federico Barcelona, Santiago Daniel Palma, María Cecilia Sánchez

**Affiliations:** ^1^ Conicet y Departamento de Ciencias Farmacéuticas, Unidad de Investigación y Desarrollo en Tecnología Farmacéutica (UNITEFA), Facultad de Ciencias Químicas, Universidad Nacional de Córdoba, Córdoba, Argentina; ^2^ Consejo Nacional de Investigaciones Científicas y Tecnológicas (CONICET), Centro de Investigaciones en Bioquímica Clínica e Inmunología (CIBICI), Córdoba, Argentina; ^3^ Departamento de Bioquímica Clínica, Universidad Nacional de Córdoba, Facultad de Ciencias Químicas, Córdoba, Argentina; ^4^ Centro de Investigación y Desarrollo en Inmunología y Enfermedades Infecciosas (CIDIE), Consejo Nacional de Investigaciones Científicas y Técnicas (CONICET), Universidad Católica de Córdoba (UCC), Córdoba, Argentina; ^5^ Departamento de Vitreo-Retina, Centro Privado de Ojos Romagosa S.A, Córdoba, Argentina

**Keywords:** doxycycline, matrix metalloproteinases, neovascularization, oxygen-induced retinopathy, pigmentary epithelium-derived factor, vascular endothelial growth factor

## Abstract

**Introduction:**

Vascular endothelial growth factor (VEGF) inhibition is currently the first-line therapy for various retinal vascular disorders, however there is a strong need to develop novel therapies to target other molecules involved in the angiogenic process. In addition to well-known antibiotic properties, Doxycycline (DXC) has versatile non-antibiotic properties, therefore, our goal was to evaluate the effect of DXC on matrix metalloproteinase-2 (MMP-2) as a potential therapeutic alternative for retinal neovascularization (NV), using vascular and glial cells and the oxygen-induced retinopathy (OIR) mouse model.

**Methods:**

MGC and BAEC viability under DXC treatment was evaluated using an MTT assay. Changes of Pro MMP-2 and MMP-2 activity were measured by gelatin zymography assay in MIO-M1 cells incubated with DXC under normoxia and hypoxic conditions. VEGF-induced angiogenesis was assessed by tube formation assay in BAEC incubated with DXC for 24 h C57BL/6 mice exposed to OIR model, were intravitreally injected with a single dose of DXC at post-natal day (P)12 and retinas evaluated at P17.

**Results:**

DXC significantly decreased pro MMP-2 and MMP-2 activity in MIO-M1 supernatants and increased hypoxic-induced mRNA expression of pigmentary epithelium-derived factor (PEDF). Moreover, DXC inhibited the VEGF-induced tube formation in endothelial cells. A single intraocular administration of DXC at postnatal day (P) 12 showed a significant decrease of pro MMP-2 and MMP-2 activity together with a reduced NV and vaso-obliteration in P17 mouse retinas of OIR eyes, while no significant difference was observed neither in MMP-2 nor in VEGF protein expression.

**Discussion:**

Our results lead to propose a possible DXC mechanism for inhibition of angiogenesis through the modulation of MMPs involving the VEGF/PEDF balance. These findings underscore the potential repositioning of DXC as a new possibility for treating ocular proliferative diseases.

## 1 Introduction

Angiogenesis plays an important role in both physiological development and pathological ocular events, being regulated by several pro-angiogenic and anti-angiogenic factors (Fogli et al., 2018). This dynamic and complex process involves basement membrane breakdown, endothelial cell proliferation and migration, extracellular proteolysis, tubulogenesis, stabilization and formation of a new basement membrane (Bishop, 2015). Several ocular disorders are associated with retinal pathologic angiogenesis, including proliferative diabetic retinopathy and retinopathy of prematurity. During pathologic angiogenesis also referred to as neovascularization (NV), a hypoxic or ischemic event, stimulates the release of pro-angiogenic factors, such as the vascular endothelial growth factor (VEGF), the main therapeutic target (Rubio and Adamis, 2015). The current treatment involves repeated intravitreal injections of VEGF-inhibitors, for instance ranibizumab (Lucentis®; Genentech, San Francisco, CA) and aflibercept (Eylea®; Regeneron Pharmaceuticals, Tarrytown, NY) (licensed to ocular route) and the off-label bevacizumab (Avastin®; Genentech, San Francisco, CA) (Amadio et al., 2016), increasing the incidence of retinal detachment, hemorrhage, cataract, endophthalmitis, patient non-compliance and cost of treatment (Joseph et al., 2017; Huang et al., 2018). Moreover, there are patients considered refractory after three monthly intravitreal anti-VEGF injections which were switched to corticosteroid treatment (Busch et al., 2018; Paz et al., 2023), considered as a second-line option.

In addition to the demand to optimize anti-VEGF therapy, it is necessary to develop novel strategies to target other factors involved in NV and its associated inflammatory process (Formica et al., 2021). In this sense, the proteolysis of extracellular matrix by matrix metalloproteinases (MMPs) —zinc-dependent endopeptidases—has been described as one of the first and most sustained activities involved in pathological angiogenesis associated with retinopathies (Sanchez et al., 2006; Sanchez et al., 2007; Rodrigues et al., 2013; Lorenc et al., 2015; Lorenc et al., 2017; Subirada et al., 2022a). Interestingly, during angiogenesis, MMPs facilitate the remodeling of the basement membrane and the extracellular matrix, allowing the migration of endothelial cells (Sapadin and Fleischmajer, 2006; Samtani et al., 2009; Cui et al., 2017).

It is well-known that the therapeutic group of tetracyclines, a broad spectrum of antibiotics, in addition to interfering with protein synthesis at the ribosomal level in bacteria, could inhibit MMPs by reducing their transcription, activation or inhibiting the active enzyme (Uitoo et al., 1994; Sobrin et al., 2000; Horwitz et al., 2014). Doxycycline (DXC) is a therapeutic tetracycline with versatile non-antibiotic properties leading to alternative uses due to anti-inflammatory, immunosuppressive, cardioprotective and anti-neoplastic effects. While the mechanisms of non-antibiotic properties are not fully known, the DXC ability to inhibit MMPs has been mainly reported (Merentie et al., 2018; Hadjimichael et al., 2020; Rok et al., 2020).

Besides the inhibition of MMPs, DXC could also regulate VEGF-A expression (Merentie et al., 2018) and promote an increase of the pigmentary epithelium-derived factor (PEDF) bioavailability (Samtani et al., 2009) in choroidal NV. Thus, DXC effect on angiogenic response could depend on the tissue type, drug exposure time and the normoxic or hypoxic conditions.

Here, we sought to contribute to further understanding of the DXC effect in both retinal cell types, from retinal environment, and oxygen-induced retinopathy mouse model (OIR), to evaluate its potential ocular use as a therapeutic alternative for retinal neovascular disorders.

## 2 Materials and methods

### 2.1 Materials

#### 2.1.1 Reagents

DXC (Doxycycline hydrochloride) and Sodium chloride (NaCl) were provided by Parafarm® (Buenos Aires, Argentina) and Ciccarelli® (Buenos Aires, Argentina), respectively. Water was supplied by Milli RO System® (Millipore®, Argentina) while reagent 3- (4,5-dimethyl-2-thiazolyl) −2,5-diphenyl2H-tetrazolium bromide (MTT), cell culture and zymography reagents were provided by Sigma-Aldrich® (Saint-Louis, MO, United States).

#### 2.1.2 Cell lines

A spontaneously immortalized human Müller glial cells line (MIO-M1) (Limb et al., 2002b), kindly provided by G. Astrid Limb (UCL Institute of Ophthalmology and Moorfields Eye Hospital, London, United Kingdom), was used. Cells were grown in Dulbecco’s Modified Eagle Medium (DMEM, Invitrogen, Buenos Aires, Argentina) with 4,500 mg/L glucose, supplemented with 2 mM L-glutamine (GlutaMAX, Invitrogen), 10% v/v of Fetal bovine serum (FBS), 50 U/mL penicillin and 50 μg/mL streptomycin (Invitrogen), at 37°C humidified atmosphere containing 5% CO_2_.

Bovine aortic endothelial cells (BAEC) were grown in DMEM medium supplemented with 20% v/v of FBS, 2 mM L-glutamine, 100 U/mL penicillin and 100 μg/mL streptomycin, at 37°C and in a humidified atmosphere containing 5% CO_2_.

#### 2.1.3 Animals

The C57BL/6J mice were acquired from the CIBICI-CONICET animal facility. Male and female of 5 months old (28–30 g) were used for retinal function and structure assessments, and P17 OIR mice (0.8–10.0 g) were used to study *in vivo* effect of DXC. A total of 36 mice were included in the study, among adult C57BL/6 mice, intravitreally injected with vehicle or DXC and P17 C^5^7BL/6 OIR mice, intravitreally injected with vehicle (OIR - Vehicle) or doxycycline (OIR - DXC). The mice were euthanized through cervical dislocation under anesthesia.

Mice were maintained under standard laboratory conditions of temperature (22°C ± 1°C) and light (12-h light/12-h dark cycle) with free access to food and water. Animals were handled according to the guidelines of the Association of Research in Vision and Ophthalmology (ARVO) Statement for the Use of Animals in Ophthalmic and Vision Research. Experimental procedures were designed and approved by the Institutional Animal Care and Use Committee (CICUAL) of the Facultad de Ciencias Químicas, Universidad Nacional de Córdoba (RD-2022–1731-E-UNC-DEC#FCQ). All efforts were made to reduce the number of animals used. No sex differences were made.

#### 2.1.4 Preparation of isotonic solutions of DXC

For *in vivo* assays, DXC solutions in concentrations ranging from 1,000 to 10,000 μg/mL were prepared with the necessary NaCl quantity to arrive at isotonic solutions. For *in vitro* assays, DXC solutions in a concentration ranging from 0.05 to 500 μg/mL were prepared from a stock DXC isotonic solution and diluted with DMEM to arrive at an isotonic and pH neutral condition.

#### 2.1.5 MIO-M1 cell supernatants

MIO-M1 cells (2 × 10^5^ cells/well) were cultured for 24 h to reach the confluence. Cells were rinsed twice with Phosphate-buffered saline (PBS), and 2 mL of DMEM-high glucose with DXC isotonic solutions (10–100 μg/mL) or vehicle was added for 24 h. Then, cell media was collected, lyophilized and suspended in 100 µL of sample buffer. The MMPs’ enzymatic activity in MIO-M1 cell supernatants treated with DXC was analyzed by gelatin zymography assays as previously described (Lorenc et al., 2015; Lorenc et al., 2017). Supernatants of MIrO-M1 cells incubated with an isotonic 0.9% NaCl solution were used as control.

### 2.2 Methods

#### 2.2.1 Cell viability assay

MIO-M1 cell viability was evaluated using the metabolic dye MTT. Briefly, 3 ×10^3^ MIO-M1 cells/well were seeded and dispersed with 100 µL of FBS medium in a 96-well plate. Then, confluent cells were treated with 100 µL of DXC isotonic solutions with concentrations ranging from 10 to 500 μg/mL. After 24 h of incubation, 10 µL of an MTT solution (5 mg/mL) was added to each well and the plate was incubated in the dark for 3 h at 37°C. The medium was then carefully removed and 200 µL of DMSO was added to solubilize the crystal violet. Optical density values were measured at 570 nm using a SpectraMax M5 plate reader (Molecular Devices, CA, United States). The results are expressed as a percentage of cell viability relative to control cells. Each concentration was evaluated by duplicating in three different experiments.

Regarding BAEC, 1.5 ×10^4^ cells/well were incubated with isotonic solutions of DXC in a concentration range of 0.05–200 μg/mL for 24 h. Then, MTT assay was carried out as described above.

#### 2.2.2 Hypoxic assay

For gas hypoxia, MIO-M1 cells were grown at 60%–70% confluence in normal conditions and transferred to a gas culture chamber (StemCell Technologies, Vancouver, BC, Canada) supplied with 1% O_2_, 94% N_2_, and 5% CO_2_. Control cells were kept in normoxia (21% O_2_). Cell experiments were conducted for 24 h, as previously described (Vaglienti et al., 2023).

#### 2.2.3 Tube-formation assay

The formation of tubules by BAEC after incubation with DXC isotonic solutions was evaluated according to a method previously described (Arnaoutova and Kleinman, 2010; Llorens de los Ríos et al., 2022). BAEC cells in the logarithmic phase and 80% confluence were placed at a density of 1.5 ×10^4^ cells/well in a 96-well “half-area” plate, previously coated with 25 µL of growth factor-reduced Corning® Matrigel free of phenol red. The cells were incubated with DXC isotonic solutions in the concentration range of 0.78–6.25 μg/mL, and VEGF (10 ng/mL) as angiogenic stimuli for 24 h (37°C and 5% CO_2_) under normoxic condition. Cells incubated with a 0.9% NaCl solution were used as control. A 30 µM sodium suramin solution (Santa Cruz Biotechnology, Texas, United States) was used as a positive inhibition control. All mentioned concentrations refer to the final concentration/well. The images were obtained using an inverted microscope (Olympus® CKX41) and were analyzed with the Angiogenesis Analyzer for ImageJ (NIH, Bethesda, MD, United States).

The tubular structures were quantified, and the percentages of angiogenesis inhibition (I%) were calculated as follows: I (%) = [1 - (total tube length treatment/total tube length control)] x 100.

#### 2.2.4 Intraocular administration of DXC in the adult mice retina

Adults C57BL/6J mice anesthetized via intraperitoneal (i.p.) injection with a solution containing ketamine (80 mg/kg)/xylazine (8 mg/kg), were intravitreally injected with 1.0 µL of a DXC isotonic solution (10,000 μg/mL) or with vehicle (0.9% NaCl) as controls. Briefly, mice were locally anesthetized with one drop of proparacaine hydrochloride 0.5% (Anestalcon, Alcon, Buenos Aires, Argentina) and eyes were punctured at the upper nasal limbus as described previously (Lorenc et al., 2017). Retinal electrophysiological studies were performed at 15- and 30- days post injection, followed by cervical dislocation euthanasia for histopathological evaluation at each point time.

#### 2.2.5 Oxygen-induced retinopathy mouse model

According to a model of OIR, previously described (Smith et al., 1994), C57BL/6J mice pups with their nursing mothers were exposed in an incubator to high oxygen concentration (75 ± 2) % between postnatal day 7 (P7) and postnatal day 12 (P12). The animals were kept in clear plastic cages with standard light cycles (12 h light/12 h dark) and oxygen was checked twice daily with an oxygen analyzer (Teledyne Analytical Instruments, CA, United States). Next, mice were returned to room air for another 5 days (relative hypoxic period, P12 - P17) and were sacrificed at P17 (peak of NV). At P12, a group of OIR mice pups were intravitreally injected into both eyes, with a DXC solution at a concentration of 10,000 μg/mL, considering intravitreal dilution (Sha and Kwong, 2006) and another group with vehicle (0.9% NaCl), used as control. Different studies were performed at P17, to evaluate MMP-2 activity, protein expression and retinal NV. On the other hand, control groups of C57BL/6J mice pups with their nursing mothers exposed to room air (RA) from P7, were injected at P12 with DXC solution at a concentration of 10,000 μg/mL, or vehicle and then, MMP-2 activity was evaluated at P17.

#### 2.2.6 Retinal cryosection preparation and protein extraction

The preparation of retinal cryosections from mice was carried out according to procedures previously described (Lorenc et al., 2017; Ridano et al., 2017; Subirada et al., 2019; Paz et al., 2020). For this purpose, eyes were enucleated and immediately fixed 2 h with freshly prepared 4% paraformaldehyde at room temperature. After that, they were incubated in 30% sucrose/PBS overnight at 4°C. Then, they were embedded in a small amount of optimum cutting temperature (Crioplast, Biopack, Buenos Aires, Argentina) compound, and 10-μm-thick radial sections were obtained. Then, retinal cryosections were stored at −20°C under dry conditions until hematoxylin-eosin staining. Optical microscopy images were obtained under a light microscope (Nikon Eclipse TE2000-E, United States).

Neural retinas were dissected from retinal pigmentary epithelium/choroid layers for Western blot or gelatin zymography assays following methods previously described (Sanchez et al., 2007; Subirada et al., 2019; Paz et al., 2020). To obtain retinal extracts, the tissue was homogenized with a lysis buffer containing 20 mM Tris-HCl, 137 mM NaCl, 2 mM Ethylenediaminetetraacetic acid (EDTA) 1% Nonidet P40, pH = 7.5, 1 mM phenylmethylsulfonyl fluoride, 2 mM sodium orthovanadate and a protease inhibitor cocktail (Sigma Aldrich, St. Louis, MO) and sonicated during 20 s at 40% of amplitude. For zymography, buffer lysis and inhibitor cocktail were prepared without EDTA and metal chelators, respectively. Retinal extracts were stored at −20°C until processed.

#### 2.2.7 Gelatin zymography assay

A 7.5% sodium dodecyl sulphate polyacrylamide gel electrophoresis (SDS-PAGE) with 1.5% gelatin (Sigma-Aldrich, St. Louis, MO), was carried out to resolve supernatants of hypoxic and normoxic MIO-M1 cells or retinal extracts of OIR mice and control mice at P17 under denaturing and non-reducing conditions as previously described (Lorenc et al., 2017). Briefly, the gels were washed for 1 h with 2.5% Triton X-100 to remove the SDS, followed by the incubation in the enzyme buffer (50 mM TrisHCl, 0.2 M NaCl, and 5 mM CaCl_2_, pH = 7.5) for 24 h at 37°C to promote MMPs enzymatic activity. After that, the gels were incubated for 30 min in 0.125% Coomassie blue R-250 and the stain was removed with the same solution without the dye. The enzymatic activity of MMPs was visualized by the proteolytic degradation of the gelatin as light bands in relation to a dark background corresponding to non-degraded gelatin. The pro MMP-2 (72 kDa) and MMP-2 (62 kDa) were identified from human capillary whole blood which was used as a positive control. The images of gels were processed, and the intensity of the bands was obtained using ImageJ software (NIH, Bethesda, MD, United States). Results are expressed as the average value of each condition.

Since there was no apparent difference between the sexes, at least three retinal extracts per group were used to carry out this assay which were prepared with at least a pool of three neural retinas of both male and female mice. On the other hand, supernatants of MIO-M1 cells were evaluated in triplicate for each condition.

#### 2.2.8 Western blot assay

The procedures were followed as previously described (Lorenc et al., 2017; Ridano et al., 2017; Subirada et al., 2019; Paz et al., 2020). For this purpose, the protein concentration of retinal extracts was determined by a Bicinchoninic Acid Protein Assay Kit (Pierce BCA, Thermo Scientific, United States) using albumin as standard. A 7.5% SDS-PAGE was carried out to resolve aliquots containing 20–30 µg of proteins which then were transferred onto nitrocellulose membranes (Amersham Hybond ECL; GE Healthcare BioSciences AB, Uppsala, Sweden). Nonspecific binding was blocked with 5% bovine serum albumin (BSA) in Tris-buffered salt (TBS) containing 0.1% Tween-20 (TBST) and the same solution was used to prepare the primary antibody. Then, membranes were incubated overnight at 4°C with the following antibodies: rabbit polyclonal anti-MMP-2 (1/1,000; Abcam, United States), mouse monoclonal anti-VEGF (1/500; R&D system) and mouse monoclonal anti-β actin or tubulin (1/2,000; Sigma-Aldrich, United States). Blots were incubated with IRDye 800 CW donkey anti-rabbit Ig and IRDye 680 CW donkey anti-mouse Ig (1/15,000 in TBS with 5% BSA) for 1 h, protected from light. After washing with TBST, membranes were visualized and quantified using the Odyssey Infrared Imaging System (LI-COR, Inc., Lincoln, NE, United States). Results are representative of at least three independent experiments.

#### 2.2.9 Quantitative real-time reverse-transcription PCR (qRT-PCR)

Total RNA was extracted from MIO-M1 cultured cells using Trizol Reagent (Invitrogen, Buenos Aires, Argentina) (Vaglienti et al., 2023). Briefly, 1 µg of total RNA was reverse-transcribed in a total volume of 20 µL using random primers (Invitrogen) and 50 U of M-MLV reverse transcriptase (Promega Corp., Madison, Wisconsin, United States). Then, cDNA was mixed with 1× SYBR Green PCR Master Mix (Applied Biosystems, Massachusetts, EE. UU.) and forward and reverse primers: VEGF-A forward CCGCAGACGTGTAAATGTTCCT and VEGFA reverse CGGCTTGTCACATCTGCAAGTA; PEDF forward: GCTGAGTTACGAAGGCGAAGT and PEDF reverse: TTGATGGGT TTGCCTGTGAT. qPCRs were carried out on an Applied Biosystems 7,500 Real-Time PCR System with Sequence Detection Software v1.4. The cycling conditions included a hot start at 95°C for 10 min, followed by 40 cycles at 95°C for 15 s and 60°C for 1 min. The specificity was verified using a melting curve analysis. The results were normalized to GAPDH: forward GATGCCCCCATGTTTGTGAT and reverse GGTCATGAGTCCTTCCACGAT. The relative gene expression was calculated according to the 2^−ΔΔCT^ method. Each sample was analyzed in triplicate. No amplification was observed in PCRs using water as a template.

#### 2.2.10 Electroretinography

According to procedures previously described (Lorenc et al., 2017; Subirada et al., 2019; Paz et al., 2020), the scotopic electroretinographic activity was recorded after treatment with DXC isotonic solution or vehicle in adults C57BL/6J mice by electroretinography (ERG). Briefly, mice were overnight (16 h) dark adapted and then manipulated under dim red illumination to accurate animal preparation and electrode placement. Mice were anesthetized via intraperitoneal (i.p.) with a solution containing ketamine (80 mg/kg)/xylazine (8 mg/kg), their pupils were topically dilated with 1% tropicamide (Mydril, Alcon, Buenos Aires, Argentina), and the cornea was lubricated with gel drops of 0.4% polyethylene glycol 400% and 0.3% propylene glycol (Systane, Alcon, Buenos Aires, Argentina) to prevent corneal damage. After body temperature stabilized at 37°C, mice were placed under a flash stimulus at a distance of 20 cm and, electrodes placed intended to measure retinal electroretinographic activity of each eye as follows: a reference electrode was inserted on the back between the ears, a grounding electrode was attached to the tail, and a gold electrode was placed in contact with the central cornea. The electroretinograms were simultaneously recorded from both eyes and ten responses to flashes of unattenuated white light (5 cd s/m^2^, 0.2 Hz) from a photic stimulator (light-emitting diodes) set at maximum brightness were amplified, filtered (1.5-Hz low-pass filter, 1,000 high-pass filter, notch activated) and averaged (Akonic BIO-PC, Argentina). Retinal responses were individually analyzed for each mouse using at least six mice per group for each assay which was carried out with both males and females, combining results, since there was no apparent difference between the sexes. At the end of the retinal electrophysiological studies mice were sacrificed by cervical dislocation.

The a-wave was measured as the difference in amplitude between the recording at the onset and trough of the negative deflection, and the b-wave amplitude was measured from the trough of the a-wave to the peak of the b-wave. The implicit times of the a- and b-waves were measured from the time of light presentation to the trough of the a-wave or the peak of the b-wave, respectively.

#### 2.2.11 Retinal whole mount staining

An assay of retinal whole-mount staining was carried out as stated previously (Lorenc et al., 2017). Briefly, OIR mice were euthanized at P17, and their eyes were enucleated and fixed with freshly prepared 4% paraformaldehyde for 2 h. The corneas were removed with scissors along the limbus, and then, the intact retinas were dissected. The retinas were blocked and permeabilized in PBS containing 5% BSA and 1% Triton-X-100 for 6 hours at 4°C. After that, retinas were incubated with Griffonia Simplicifolia Isolectin IB4 Alexa fluor-488 conjugated (GSA-IB4, 1/100; Molecular Probes, Eugene, OR, United States) overnight at 4°C. Retinas were then washed with TBS containing 0.1% Triton-X-100. Flats were examined by confocal laser-scanning microscopy Olympus FV1200 (Olympus FluoView FV1200, Japan). Each image was the flattened result of 10 photos (10-μm-thick each section) taken at plane z. Immunostaining was quantified as fluorescence intensity labelling (% area) from three different whole mounts of each experimental group (n = 3) using ImageJ Fiji software (National Institutes of Health, Bethesda, MD, United States).

#### 2.2.12 Statistical analysis

Statistical analysis was performed using the GraphPad Prism 7.0 software. A p-value <0.05 was considered statistically significant. Parametric or non-parametric tests were used according to the variance homogeneity evaluated by F or Barlett’s tests, and Kruskal–Wallis followed by Dunn’s multiple comparisons post-test or one-way or two-way ANOVA followed by Bonferroni multiple comparisons post-test, or two-tailed unpaired t-test, as appropriate. Data represents the mean ± standard error of the mean (SEM) or the median with the interquartile range depending on parametric or non-parametric test, respectively.

## 3 Results

### 3.1 DXC modifies cell viability in a concentration-dependent manner

**FIGURE 1:**
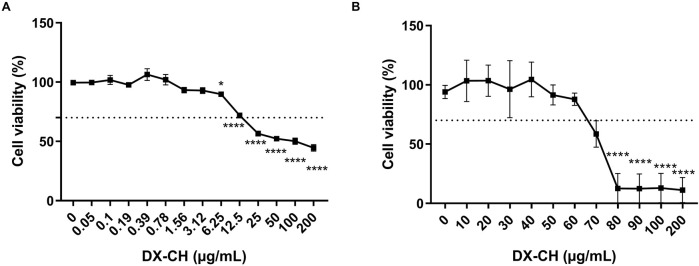
Effect of DXC on cell viability in **(A)** BAEC and **(B)** MIO-M1 cells. MTT assay represents the percentage of viable cells treated with DXC for 24 h compared to control cells. Data represent mean ± SEM, analyzed using one-way ANOVA followed by Bonferroni post-test. *p < 0.05; ****p < 0.0001. Results represent three independent experiments performed in duplicated.

Previous studies have demonstrated that although Müller glial cells (MGCs) are more sensitive to changes in the microenvironment, they are highly resistant cells to stimuli (Coughlin et al., 2017; Subirada et al., 2018; Pereiro et al., 2024). Cell viability under DXC treatment in BAEC and MIO-M1 cell lines was assessed by MTT assay. Exposition time to DXC was 24 h, and the concentrations tested ranged from 0.05 to 200 μg/mL and 10–500 μg/mL for BAEC and MIO-M1 cells, respectively (Figure 1). Results showed that the percentage of viable cells after 24 h of exposure decreased proportionally to the drug concentration in both cell lines, and no significant differences were observed compared to control (0 μg/mL DXC) when the cells were treated with DXC isotonic solutions at concentrations up to 3.12 μg/mL and 70 μg/mL for BAEC (Figure 1A) and MIO-M1 cells (Figure 1B), respectively. Although DXC isotonic solution at 6.25 μg/mL presented significant differences compared with control, only a decrease of 10% in the cell viability was observed.

### 3.2 DXC inhibits enzymatic activity of pro MMP-2 and MMP-2 in MIO-M1 cells supernatant

Given that MMP-2 participates in the regulation of retinal NV (Sanchez et al., 2006; Rodrigues et al., 2013; Lorenc et al., 2017) and that MGCs under normal conditions spontaneously secrete MMPs to the culture medium (Limb et al., 2002b; Limb et al., 2002a) we evaluated the effect of DXC on MMP-2 activity in MIO-M1 cells under normoxia (21% O_2_) or hypoxia (1% O_2_). For this purpose, we incubated MIO-M1 in the presence or absence of 10 or 50 μg/mL of DXC for 24 h and then analyzed the supernatants by gelatin zymography assay.

**FIGURE 2:**
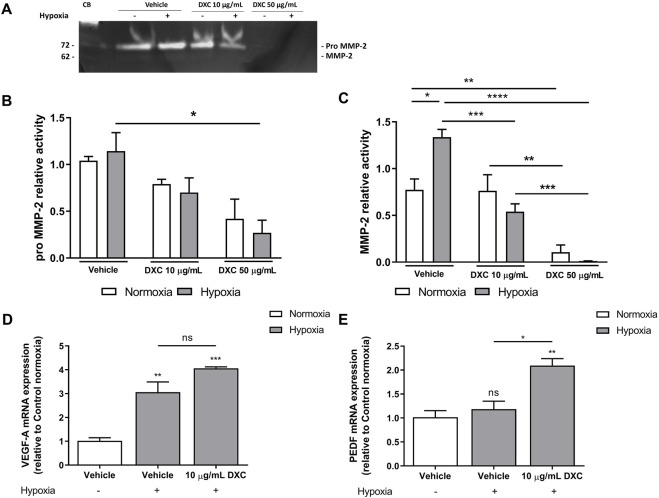
Evaluation of DXC effect in MMP-2 activity, VEGF mRNA and PEDF mRNA in MIO-M1 cells. **(A)** Representative gelatin zymography assay of MIO-M1 culture supernatants obtained from cells incubated with 10 and 50 µg/mL of DXC for 24 h in normoxic or hypoxic conditions. Capillary blood (CB) was used as a gelatinase control of pro MMP-2 (72 kDa) and MMP-2 (62 kDa). Bars represent the average of enzymatic activity of **(B)** pro MMP-2 and **(C)** active MMP-2, which were quantified by densitometric analysis and expressed as enzymatic activity relative to control (vehicle) in normoxic (white bars) and hypoxic (grey bars) conditions. Bars represent the average of **(D)** VEGF-A and **(E)** PEDF mRNA levels relative to β-actin in MIO-M1 cell culture lysates, after 24 h of incubation with 10 μg/mL of DXC or vehicle in hypoxic (grey bars) condition, relative to control in normoxic (white bars) condition. Data represent mean ± SEM. One-way ANOVA, Bonferroni post-hoc were carried out (*p < 0.05; **p < 0.01, ***p < 0.001, ****p < 0.0001, ns = non-significant). Three independent assays were carried out and each condition was tested in duplicate.

As expected, levels of enzymatic activity corresponding to pro MMP-2 (72 kDa) together with a minor presence of the active MMP-2 form (62 kDa), were detected in supernatants of MIO-M1 cells incubated 24 h with vehicle in normoxia (Figures 2A–C), according to previous studies (Limb et al., 2002a; Barcelona et al., 2013; Lorenc et al., 2015). As evidence of the hypoxic treatment, a significant increase in MMP-2 activity was observed in vehicle group (Figures 2A, C). Exposing MIO-M1 cells to both DXC concentrations did not show statistically significant changes in pro MMP-2, nor in MMP-2 activity, between normoxic and hypoxic conditions. In normoxia, treatment with 50 µg/mL of DXC efficiently inhibited ∼90% MMP-2 activity compared to vehicle, and MMP-2 activity was significantly different between DXC treatment at both concentrations (Figures 2A, C). In hypoxic conditions, pro MMP-2 activity showed a significant reduction of ∼80% in supernatants of MIO-M1 cells treated with 50 μg/mL of DXC respect to vehicle, and MMP-2 activity showed a significant decrease of 60% and of almost 100%, when the cells were treated with DXC 10 µg/mL and 50 µg/mL, respectively (Figures 2A–C). Altogether, these results showed that the inhibition of pro MMP-2 and MMP-2 enzymatic activity by DXC was concentration-dependent.

### 3.3 DXC increases PEDF mRNA expression in hypoxic MIO-M1 cells culture

Under hypoxic conditions, MGCs increase VEGF retinal levels, the main pro-angiogenic factor involved in angiogenesis (Wang et al., 2010) and led to the upregulation of PEDF to restrict the vascular growth (Yang et al., 2012). To determine if trophic factors levels were modified by DXC treatment, we further quantified the transcript levels of VEGF and PEDF in hypoxic MIO-M1 cells (Figures 2D, E). Results showed a significant increase in VEGF mRNA levels in hypoxia at least 4 times higher than in normoxia, but no significant differences were shown with DXC treatment (Figure 2D). By contrast, an increase in the PEDF mRNA expression was observed in hypoxic MIO-M1 cells treated with DXC (Figure 2E). In summary, the results indicate that DXC directly modulates PEDF/VEGF balance in hypoxic MGCs suggesting an anti-angiogenic cell response.

### 3.4 DXC reduces endothelial cell tube formation

**FIGURE 3:**
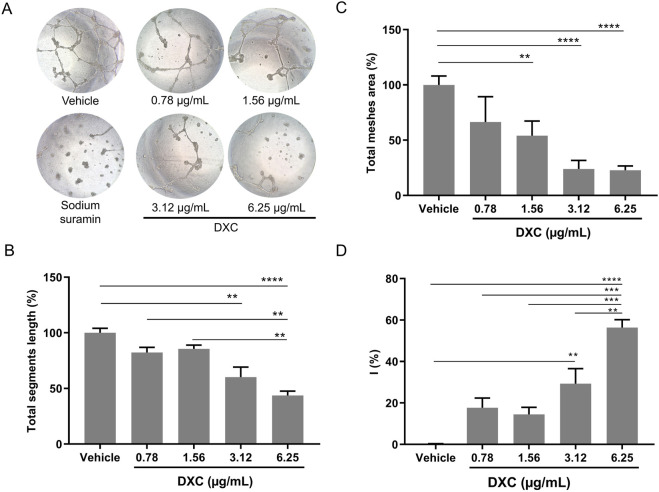
Analysis of tube formation in BAEC in response to different concentrations of DXC. **(A)** Representative bright field microscopy images of BAEC (≈1.5 × 10^4^ cells) on Matrigel with VEGF (10 ng/mL) incubated with vehicle (negative control), DXC (0.078–6.25 µg/mL) or sodium suramin (positive control of inhibition). **(B)** Quantitative analysis in terms of the total segment length and **(C)** average mesh area and their ratios are represented in the bar graphs expressed in percentage (%) in relation to vehicle. **(D)** Percentages of inhibition (I%) in relation to vehicle, were calculated as follows: I (%) = [1 − (Total tube length in treatment/Total tube length in control)] × 100. Data represent mean ± SEM. One-way ANOVA, Bonferroni post-hoc were carried out (**p < 0.01; ***p < 0.0001, ****p < 0.0001). Three independent assays were carried out and each condition was tested in triplicate.

Endothelial cell migration is essential to angiogenesis during proliferative retinopathies (Rodrigues et al., 2013). Therefore, a tube formation assay was carried out to evaluate the effect of DXC on the formation of vascular structures since this assay comprises different stages of the angiogenic process. For this purpose, we seeded BAECs in Matrigel and treated them with vehicle or DXC, both in the presence of VEGF (10 ng/mL). DXC induced a significant reduction in tube formation compared to control (Figure 3A).

Quantitative analysis showed that DXC significantly decreased the tube length (Figure 3B) in cells treated with 3.12 and 6.25 µg/mL, and the average of total mesh area (Figure 3C) with 1.56, 3.12 and 6.25 µg/mL DXC, both compared with control. Thus, the percentage of angiogenesis inhibition was greater in cells treated with high drug concentration showing a dose-dependent effect of DXC (Figure 3D). These changes were not observed with the vehicle. Considering that the drug concentrations 1.56 and 3.12 µg/mL did not significantly affect the cell viability of BAEC (Figure 1A), in comparison with the vehicle, and only a 10% of cell viability reduction was observed at 6.25 µg/mL, the antiangiogenic effect of DXC on tube formation was demonstrated. In addition, greater isolated fragments were observed in cells incubated with 30 µM sodium suramin used as a positive control of angiogenesis inhibition.

### 3.5 DXC maintains electrophysiology and histological structure of the retina

Before administration in OIR mice, the effects of intravitreally injected DXC on retinal function and structure in adult C57BL/6J mice were evaluated. The functional status of the retinas, analyzed by scotopic ERG, and the histopathological evaluation were performed 15 and 30 days after a 1.0 µL intravitreal injection of DXC isotonic solution (10,000 μg/mL). This was done to test a drug dose of 10 µg and achieve a drug concentration of 1 μg/mL inside the eye, considering the mouse vitreous volume.

**FIGURE 4:**
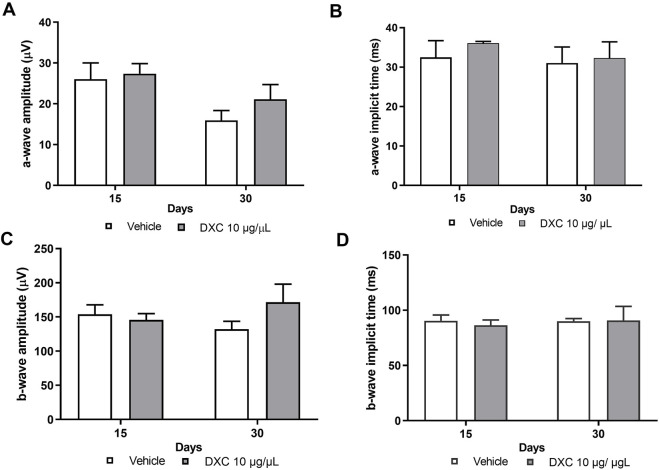
Scotopic ERG in adult mice. Bars represent the average of **(A)** a-wave amplitudes, **(B)** a-wave implicit times, **(C)** b-wave amplitudes and **(D)** b-wave implicit times, recorded at 15 and 30 days post intravitreal injection of 10 μg/μL DXC in adult C57BL/6J mice. Data represent mean ± SEM. No significant differences were obtained from statistical analysis. Two-way ANOVA, Bonferroni post-test was carried out (analyzed as a repeated measure, n = 6).

No significant differences in a-wave and b-wave amplitudes were observed in mice injected with DXC compared to the vehicle group (Figures 4A, C). Also, implicit times of the a- and b-waves showed a similar pattern as a vehicle group (Figures 4B, D).

**FIGURE 5:**
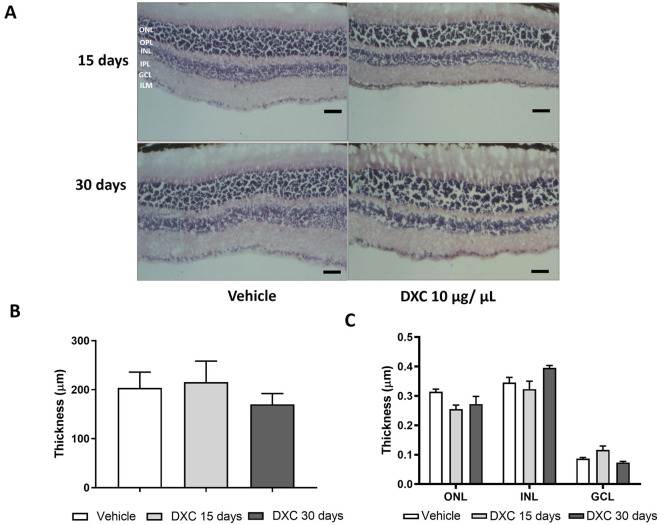
Histological evaluation of DXC effect on adult mice retinas. **(A)** Representative photomicrographs of 10 µm sections of retinal tissue stained with hematoxylin-eosin from adult C57BL/6J mice at 15 and 30 days after intravitreal injection of 10 μg/μL of DXC. **(B)** Total retinal thickness and **(C)** Retinal thickness of inner nuclear layer (INL), ganglion cell layer (GCL) and outer nuclear layer (ONL). Outer plexiform layer (OPL), inner plexiform layer (IPL). Data represent mean ± SEM. No significant differences were obtained from statistical analysis. Two-way ANOVA, Bonferroni post-test was carried out (n = 3). Scale bar 50 μm, 200× of magnification.

Furthermore, retinal thickness analysis revealed no differences in the entire retina (Figure 5B), nor in the different layers (Figure 5C) of DXC-treated mice with respect to vehicle group. These results clearly showed that DXC did not alter the retinal function or histological morphology.

### 3.6 DXC decrease NV in oxygen-induced retinopathy mouse model

To evaluate the *in vivo* efficacy of DXC in retinal NV, groups of newborn mice exposed to the OIR model were intravitreally injected at P12 with 1 µL of an isotonic drug solution (10,000 µg/mL) for testing a dose of 10 μg, considering the intravitreal dilution. Then, the ocular tissue was removed at P17 to analyze vascular alterations in whole-mounted retinas, enzymatic activity and expression of MMP-2, in retinal extracts.

**FIGURE 6:**
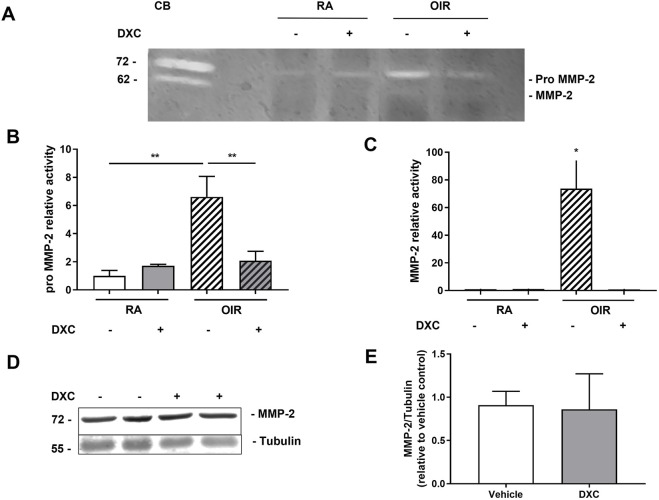
DXC effect on MMP-2 activity and protein expression in OIR mouse model. **(A)** Representative gelatin zymography assay showing enzymatic activity of pro MMP-2 and MMP-2 in retinal extracts samples of RA and OIR mice, intravitreally injected with vehicle or DXC (10 µg/µL). Bars represent the average of enzymatic activity of **(B)** pro MMP-2 and **(C)** active MMP-2, quantified by densitometric analysis and expressed as enzymatic activity relative to vehicle in RA condition. Data represent mean ± SEM. Two-way ANOVA, Bonferroni post-hoc were carried out, at least n = 3 mice in each group. **(D)** Representative blot of MMP-2 and Tubulin, as a loading control, from neural retina extracts of OIR mice, intravitreally injected with vehicle or DXC (10 µg/µL). **(E)** Bars represent the average levels of protein expression of MMP-2, relative to vehicle, from neural retina extracts of OIR mice intravitreally injected with vehicle or DXC (10 μg/μL), quantified and normalized to Tubulin. Data represent mean ± SEM. Two-tailed unpaired t-test, *p < 0.05; **p < 0.01, n = 3 mice in each group.

Gelatin zymography assay showed that the pro MMP-2 activity significantly increased at P17 OIR compared to RA mice in retinal extracts of eyes injected with vehicle (Figures 6A, B). In addition, a significant decrease of both pro MMP-2 and MMP-2 enzymatic activity was observed in retinal extracts of mice OIR injected with DXC with respect to vehicle (Figures 6A, C), while no significant differences were observed in MMP-2 protein expression between OIR vehicle or DXC treated mice (Figures 6D, E).

**FIGURE 7:**
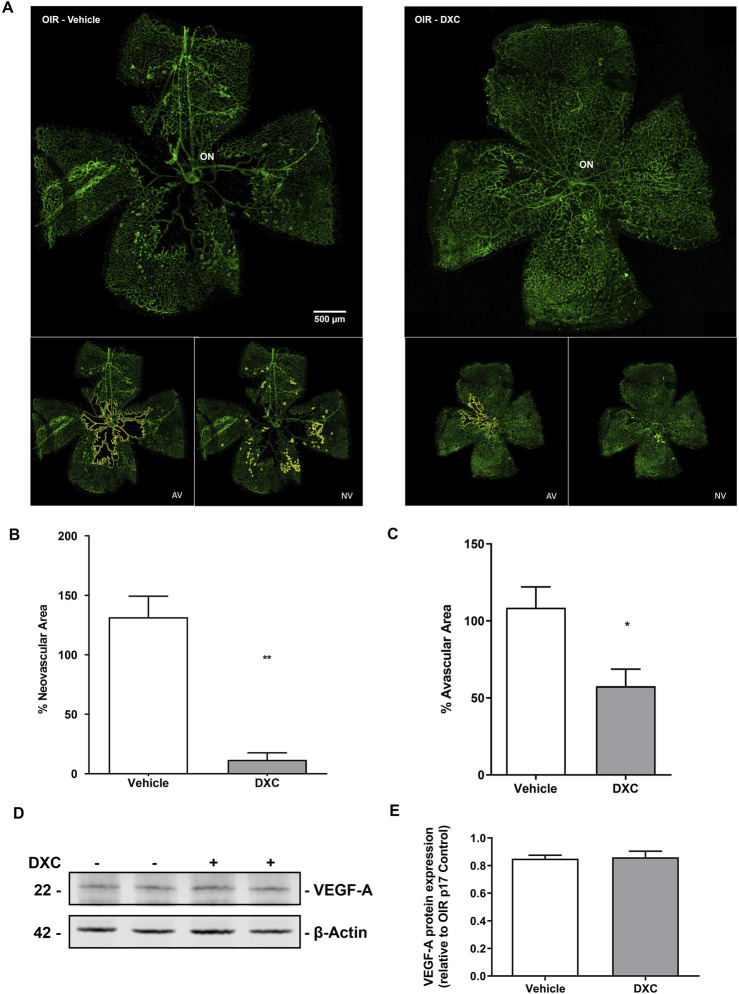
Analysis of DXC effect in OIR mouse model. **(A)** Representative microphotographs of whole retina labeled with Alexa Fluor-488 - conjugated GSA-IB4 from OIR mouse intravitreally injected with vehicle or DXC (10 μg/μL). The avascular area (AV, left inset of figure A in RA and OIR conditions) and the area occupied by neovascularization tufts (NV, right inset of figure A in RA and OIR conditions) were outlined in yellow. **(B)** Bars represent the average of NV tufts area %, quantified as a percentage of the whole retinal area. **(C)** Bars represent the average of avascular area %, quantified as a percentage of the central avascular area to the whole retinal area. Data represent mean ± SEM (*p < 0.05; **p < 0.01, n = 3). Scale bar 500 μm, ×100 of magnification. ON: optic nerve. **(D)** Representative blot of VEGF-A and β-actin, as a loading control, from neural retina extracts of OIR mice, intravitreally injected with vehicle or DXC (10 μg/μL). **(E)** Bars represent the average levels of protein expression of VEGF-A from neural retina extracts of OIR mice, intravitreally injected with vehicle or DXC (10 μg/μL), quantified as optic density, normalized to β-actin and relativized to vehicle group. Two-tailed unpaired t-test, n = 3 mice in each group.

A staining of whole-mounted retinas assay (Figure 7A) was performed to determine if the inhibition of the MMP-2 enzymatic activity observed in both, *in vitro* and *in vivo* assays, was able to reduce the vascular alterations in the OIR mouse model. A single intraocular injection of DXC administered at P12 in OIR mice produced an improvement in the vascular alterations observed in P17 OIR mouse retinas injected with vehicle (Figures 7A–C). Both the amount of neovascular tufts (Figure 7B) and the avascular area (Figure 7C) at P17 OIR, were significantly lower after the DXC treatment, respect to mice injected with vehicle. Finally, OIR mice groups showed similar VEGF-A protein expression at P17 (Figures 7D, E).

## 4 Discussion

Retinal pathological NV involves complex interactions of a variety of angiogenic factors, mainly VEGF, as well as MMPs participation and is a leading cause of serious vision loss. Several studies have demonstrated a significant reduction in NV in some -but not all-patients with retinopathies receiving anti-VEGF therapy (Ip, 2012; Bressler et al., 2013; Gross et al., 2015). Therefore, there is an unmet need to develop new alternative therapeutics in the field of ocular angiogenesis. In this work, we investigated the effect of DXC in retinal pathological NV using *in vitro* assays and the established *in vivo* OIR model.

DXC is an inexpensive drug used safely in pharmacotherapy as an antibiotic, and it has been proposed as an MMPs inhibitor, given the effects observed in animal models of corneal or choroidal NV. In this sense, eye topical treatment with DXC showed that it contributed significantly to preventing corneal angiogenesis and inflammation (Ling et al., 2013), as well as promoted corneal healing and reduced opacity (Yi and Zou, 2019) in murine alkali-burned corneas. Additionally, DXC decreased gelatinolytic activity and expression of MMP-1, MMP-9 and MMP-13 (Bian et al., 2016). In other ocular tissues, DXC administered into drinking water, induced less NV in terms of blood vessel volume in a murine model of choroidal NV, a reduction in blood vessel growth and migration directed *in vivo* angiogenesis assay (DIVAA) and the regression of pterygium cell lesions in a mouse model (Samtani et al., 2009; Cox et al., 2010). Intraperitoneal injections of DXC, also inhibited leakage from choroidal NV, suppressed fibrosis and decreased the ratio of fibrotic/NV area (Peng et al., 2018). Furthermore, a dose of 200 mg/day DXC for 4 months, as co-treatment of intraocular bevacizumab was able to control active wet age-associated macular degeneration like standard treatment, reducing the total number of intraocular bevacizumab injections (Mirshahi et al., 2017). Although these different experimental studies have aimed to evaluate the effect of DXC in some ocular disorders, little is known about its effects on proliferative retinopathies.

Here, we first analyzed cell viability in MGCs and BAECs to determine the highest non-cytotoxic concentration of DXC to be selected for *in vitro* and *in vivo* studies. As was previously stated, although MGCs are more sensitive to changes in the microenvironment, they are highly resistant cells to harmful stimuli (Subirada et al., 2018). Consistent with previous observations, the MIO-M1 cells did not show a statistically significant reduction in cell viability up to 70 μg/mL (Salimiaghdam et al., 2022), while in the same culture conditions, BAEC were sensitive to approximately 10 times lower concentrations.

In the retina, both MGCs and endothelial cells are MMPs-producing cells (Miyata et al., 2012; Rodrigues et al., 2013; Lorenc et al., 2015). Basal MMP-2 expression has been described in healthy adult mice retinas (Lorenc et al., 2017), and under normoxia in the supernatants of human MGCs culture (Limb et al., 2002a; Lorenc et al., 2015). Considering that MMP-2 has been associated with pathological NV observed in proliferative retinopathies, we decided to evaluate the DXC effect on the gelatinase activity of MMP-2 by zymography. Quantitative analysis revealed a significant decrease in pro MMP-2 activity with a dose of 50 μg/mL DXC under hypoxic conditions, being this effect more evident with the rising DXC concentration (10–50 μg/mL) on the MMP-2 activity suggesting that the inhibition of both pro MMP-2 and MMP-2 enzymatic activity by DXC under hypoxia was concentration-dependent. It could suggest that DXC may act as a non-competitive inhibitor of MMPs, probably by interacting with the zinc or calcium atoms within the structural center of these enzymes (García et al., 2005; Samtani et al., 2009; Bian et al., 2016).

Knowing that the balance towards the anti-proliferative effect can be shifted by increasing the synthesis of antiangiogenic proteins, such as PEDF, or by decreasing the synthesis of the pro-angiogenic proteins, mainly VEGF (Yafai et al., 2007; Medina-Arellano et al., 2024), we decided to evaluate the changes in the VEGF and PEDF mRNAs. Our results showed an increase in the hypoxic-induced mRNA expression levels of PEDF in MIO-M1 cells incubated with DXC, while VEGF transcript was not modified demonstrating an anti-angiogenic effect mediated by MGCs response.

In endothelial cells, it has been previously demonstrated that DXC significantly decreased MMP-2 and MMP-9 enzymatic activity in the presence of VEGF (Su et al., 2013). Here, we analyzed whether the DXC treatment could have a direct impact on angiogenesis using an endothelial cell tube formation assay. Our results demonstrated that DXC strongly inhibited the formation of tubular structures in a dose-dependent manner. This could be related to the inhibition of MMPs enzymatic activity or, even to the decrease of basal VEGF-A expression induced by DXC in endothelial cells, as it has been observed in a previous study using a similar concentration range from 0.010 to 1 μg/mL (Merentie et al., 2018).

In this study, we emphasize the significance of the *in vitro* findings by analyzing the effects of DXC on the retinal activity and expression of MMP-2 in a mouse model of OIR which reproduces neovascular disorders such as retinopathy of prematurity and proliferative diabetic retinopathy (Smith et al., 1994). Initially, we evaluated the ocular toxicity of intravitreally injected DXC in healthy retinas from adult C57BL/6J mice. We demonstrate that a single intraocular injection of DXC did not change the electrical activity or histological structure of the retina, indicating absence of damage and of ocular toxicity. In the OIR mouse model, an increase in VEGF levels promotes the expression and enzymatic activity of MMP-2, which leads to retinal NV (Rodrigues et al., 2013). In fact, the VEGF protein levels increase after the hypoxic event, peaking at P17. At the same time, the MMP-2 expression and enzymatic activity were also increased, which we have previously demonstrated. Moreover, we found a close association between the MMP-2 active levels at different stages of the OIR and the development of NV, which provides a clear picture of the role of MMP-2 in this pathological process (Lorenc et al., 2017). In the present work, after DXC treatment, we demonstrated a decreased enzymatic activity of MMP-2 in retinal extracts at P17 OIR, without an evident modification in the protein expression, suggesting the *in vivo* regulation of both pro and MMP-2 active levels. In line with these results, DXC effectively prevented the abnormal NV and significantly enhanced the physiological revascularization of the retinal vascular plexus at P17 OIR.

It has been previously proposed that MMPs can regulate extracellular VEGF bioavailability by cleaving matrix-bound isoforms of VEGF and releasing soluble active fragments. In contrast, the main VEGF ocular antagonist PEDF is proteolyzed by MMP-2 and MMP-9, which can abolish PEDF’s anti-angiogenic properties (Lee et al., 2005). In this regard, the observed decrease in MMP-2 gelatinase activity in retinal extracts of OIR mice, after DXC treatment, may impair the positive feedback between MMP-2 and VEGF, and favor an increase in PEDF levels, restraining retinal NV. A similar effect has been reported in an experimental choroidal NV rat model showing that DXC effectively shifted the balance between VEGF/PEDF factors to inhibit the progression of NV (Samtani et al., 2009). In a previous study, we have described the anti-angiogenic and anti-gliotic effects in MGCs under hypoxia by antibiotic-related drugs (e.g., rapamycin) (Subirada et al., 2022b) which also affected angiogenic regulators.

Our study constitutes the first evidence of the inhibitory effect of DXC on MMP-2 enzymatic activity in both, *in vitro* and *in vivo* models, in addition to its anti-angiogenic property in proliferative retinopathies. The present results lead us to propose a possible mechanism for DXC’s inhibition of NV through the modulation of MMPs that affects the VEGF/PEDF balance. This potential repositioning of DXC highlights its clinical relevance. Further studies should be conducted to evaluate if DXC holds promise as a new co-treatment with anti-VEGF agents for retinal NV, utilizing sub-therapeutic antibiotic doses as in this work.

## Data Availability

The original contributions presented in the study are included in the article, further inquiries can be directed to the corresponding author.

## Abbreviations

DXC, Doxycycline; MGCs, Müller glial cells; MMPs, Matrix metalloproteinases; MMP-2, Matrix metalloproteinase-2; NV, Neovascularization; OIR, Oxygen-induced retinopathy; PEDF, Pigmentary epithelium-derived factor; RA, Room Air; VEGF, Vascular endothelial growth factor.
